# Validation of Synthetic Megavoltage Computed Tomography (MVCT) for Dose Calculation in Radiotherapy Treatment Planning

**DOI:** 10.3390/cancers18101603

**Published:** 2026-05-14

**Authors:** Aurora Corso, Niki Martinel, Mubashara Rehman, Joseph Stancanello, Christian Micheloni, Cristian Deana, Cristina Cappelletto, Paola Chiovati, Riccardo Spizzo, Giuseppe Fanetti, Andrea Dassie, Michele Avanzo

**Affiliations:** 1 Medical Physics Department, Centro di Riferimento Oncologico di Aviano IRCCS, 33081 Aviano, Italy; aurora.corso@cro.it (A.C.); cristian.deana@studenti.units.it (C.D.); ccappelletto@cro.it (C.C.); pchiovati@cro.it (P.C.); adassie@cro.it (A.D.); 2 Scuola di Specializzazione di Fisica Sanitaria, Università di Padova, 35122 Padova, Italy; 3 Machine Learning and Perception Lab, Università degli Studi di Udine, 33100 Udine, Italy; niki.martinel@uniud.it (N.M.); rehman.mubashara@spes.uniud.it (M.R.); christian.micheloni@uniud.it (C.M.); 4 Elekta SA, 92100 Boulogne-Billancourt, France; joseph.stancanello@elekta.com; 5 Molecular Oncology Division, Centro di Riferimento Oncologico di Aviano IRCCS, 33081 Aviano, Italy; rspizzo@cro.it; 6 Radiation Oncology Department, Centro di Riferimento Oncologico di Aviano IRCCS, 33081 Aviano, Italy; giuseppe.fanetti@cro.it

**Keywords:** synthetic images, metal artifact reduction, deep learning, megavoltage CT, head and neck radiotherapy, dose calculation, quality assurance

## Abstract

**Background/Objectives**: Dental metallic implants cause severe streaking artifacts in kilovoltage CT (kVCT), compromising dose calculation in radiotherapy (RT) treatment planning. The purpose of this study is to assess the dosimetric agreement of synthetic MVCT (sMVCT) images generated from artifact-affected kVCT using a deep learning network with respect to true MVCT (tMVCT) acquired at the treatment machine. **Methods**: Nineteen head and neck cancer patients with dental metallic implants treated with RT were included. Planning kVCT images were converted to sMVCT using Metal Artifact Reduction through Domain Transformation Network (MAR-DTN), a UNet-inspired deep learning network. The sMVCT images were rigidly registered to true MVCT (tMVCT) acquired on the Hi-Art II Tomotherapy system. Mean Hounsfield Unit (HU) values were compared across seven structures (thyroid, bilateral parotids, brainstem, spinal cord, GTV, PTV70) using pairwise Wilcoxon tests and Two One-Sided Tests (TOST) for statistical equivalence within a pre-specified margin of ±20 HU (corresponding to a 2% deviation in physical density). Dose distributions were recalculated on sMVCT using the AAA algorithm and compared to reference tMVCT-based plans via dose–volume histogram (DVH) metrics, evaluated for equivalence by TOST within a margin of ±2% of the prescribed dose (±142 cGy of 70.95 Gy), and via 3D gamma index, evaluated by one-sided non-inferiority test against the clinically accepted thresholds of 90% (2 mm/2%) and 95% (3 mm/3%). A pre-specified sensitivity analysis was performed by repeating all comparisons on the strictly independent sub-cohort (*n* = 16) excluding three patients drawn from the MAR-DTN training set. **Results**: All seven anatomical structures showed statistical equivalence between sMVCT and tMVCT under the ±20 HU margin (TOST *p* < 0.05; mean HU differences in the range −1.1 to +8.4 HU; all Wilcoxon *p* > 0.05). All nine DVH metrics achieved formal dosimetric equivalence within ±2% of the prescribed dose (TOST *p* < 0.05). Mean 3D gamma pass rates were 94.3% (95% CI: 89.3–97.1) for the 2 mm/2% criterion and 97.6% (95% CI: 94.8–99.0) for the 3 mm/3% criterion, both formally non-inferior to the respective clinical thresholds (*p* < 0.0001). Residual gamma failures were concentrated at the patient surface, consistent with inter-session repositioning uncertainty rather than errors in synthetic image generation. Sensitivity analysis on the *n* = 16 sub-cohort confirmed all conclusions, with mean HU and DVH differences smaller than in the full cohort for the structures showing the largest mean differences, and comparable for the remaining structures, with all TOST equivalence and gamma non-inferiority tests confirmed in both cohorts. **Conclusions**: sMVCT images generated via MAR-DTN show dosimetric agreement with physically acquired tMVCT in head and neck patients with dental implants, formally demonstrated by TOST equivalence within ±2% of prescribed dose for all DVH metrics. The combined HU and gamma index framework presented here represents a promising quality assurance approach for AI-based synthetic imaging tools in radiotherapy, pending validation in larger prospective multicentre cohorts.

## Simple Summary

Dental metallic implants cause severe streaking artifacts in kilovoltage computed tomography, compromising dose calculation in radiotherapy treatment planning. Artifact-free synthetic megavoltage computed tomography images can be generated via Metal Artifact Reduction through Domain Transformation Network, a deep learning neural network. In this study, we demonstrate that the resulting synthetic images show close dosimetric agreement with the true megavoltage images, formally confirmed by equivalence testing within ±2% of the prescribed dose. These results support the dosimetric feasibility of synthetic megavoltage images as a promising alternative to true megavoltage imaging for radiotherapy planning in patients with dental implants, pending validation in larger prospective cohorts.

## 1. Introduction

Radiation therapy (RT) is one of the most common treatment modalities for cancer, valued for its clinical effectiveness and favorable toxicity profile. Its primary goal is to deliver a high radiation dose to the target volume—i.e., the tumor—while minimizing exposure to surrounding healthy structures, known as organs at risk (OARs). This is achieved through a dedicated treatment planning phase, in which dose constraints are optimized to balance tumor control and normal tissue sparing [1,2,3,4].

Medical imaging plays a central role in this process. CT imaging, acquired using a kilovoltage (kV) X-ray beam to maximize soft tissue contrast while keeping the imaging dose to a minimum, provides the means to delineate the target and OARs [3]. A key advantage of CT is its ability to provide Hounsfield Unit (HU) values, which encode the X-ray attenuation of each voxel relative to water. HU values are converted into electron density via a scanner-specific HU-to-electron-density calibration curve, providing the three-dimensional tissue density map required for accurate dose calculation and plan optimization [1]. When the RT treatment is delivered, the patient is re-imaged immediately prior to each fraction and its positioning is adjusted to match that of the planning CT, a technique called image-guided radiotherapy (IGRT) [5]. To date, the most widely adopted imaging technique for IGRT is kilovoltage CBCT (kV-CBCT). A variant based on a megavoltage (MV) beam—megavoltage CT (MVCT)—has also been introduced, leveraging the treatment beam itself to image the patient prior to delivery by means of an MV beam [6]. Importantly, MVCT provides relative electron-density maps that can be directly used for dose calculation and treatment planning, with studies demonstrating dosimetric accuracy comparable to that of kV-CT [5].

The most striking advantage of MVCT is being free from artifacts from metal implants such as dental and hip prostheses and pelvic/abdominal clips which largely affect CT and kV-CBCT. Metal artifacts primarily manifest as streaking patterns on CT and kV-CBCT images arising from dental restorations [7] or other metallic implants. Streaking artifacts can significantly hinder the visualization of target and OARs. Metal artifacts occur because the attenuation coefficient of metal in the diagnostic X-ray spectrum is much greater than soft tissue and bone [8]. Their presence introduces substantial uncertainties in the electron-density map. Moreover, the Hounsfield unit (HU) values of metallic materials are strongly dependent on the CT energy spectrum, further complicating accurate density assignment and dose calculation [9]. MVCT is intrinsically less affected by metal artifacts, as MV X-rays undergo less pronounced attenuation differences across metal and soft tissue [10]. kV-CT, as already widely applied in diagnostic images, and kV-CBCT can be corrected for metal artifacts by means of the so-called Metal Artifact Reduction (MAR) mostly based on linear in-painting algorithms [11]. These approaches estimate the region in the projections from the sinogram that are affected by beam blocking due to the metal implants and interpolate the missing information to create a streak-free tomographic image. Linear in-painting tries to restore the missing information in the sinogram based on neighboring information but can also introduce distortion in the HU value due to the interpolative nature.

Deep learning approaches for synthetic CT generation have been extensively investigated in radiotherapy. Generative adversarial networks (GANs) and UNet-based architectures have been applied to generate synthetic kVCT from MRI for MRI-only planning [12], and synthetic CT from cone-beam CT (CBCT) for adaptive radiotherapy workflows [13]. In the specific context of metal artifact reduction, prior approaches have used MVCT [14] or megavoltage CBCT (MVCBCT) [15] as prior images in sinogram-based iterative algorithms to correct kVCT artifacts, achieving clinically acceptable dosimetric accuracy. However, these methods require access to an MVCT acquisition and do not generate a standalone synthetic image suitable for direct use in treatment planning. Deep learning-based domain transformation—converting artifact-affected kVCT directly into sMVCT—represents a more recent and clinically flexible approach.

Our research group has previously developed and published a deep learning network—MAR using Domain Transformation Network (MAR-DTN)—to convert artifact-affected kVCT into artifact-free synthetic MVCT (sMVCT) for treatment planning and dose calculation [16]. The purpose of this study is to compare HU values and dose distributions calculated on sMVCT against those obtained on tMVCT acquired at the treatment machine, with the aim of establishing a quality assurance framework for the clinical use of synthetic images in radiotherapy treatment planning. To the best of our knowledge, this is the first study to validate the dosimetric accuracy of sMVCT images—generated from artifact-affected kVCT via a deep learning domain transformation network—against physically acquired tMVCT for radiotherapy treatment planning in head and neck patients with dental metallic implants.

## 2. Materials and Methods

### 2.1. Patient Dataset

Nineteen patients treated with Intensity-Modulated RT (IMRT) at the Centro di Riferimento Oncologico di Aviano (CRO) IRCCS for head and neck cancer were included in this study. This study was conducted as part of two protocols approved by the Regional Ethics Committee (CRO-2017-50; CRO-2019-66). All clinical data were collected and processed under applicable ethics approvals. Written informed consent was obtained from all patients.

Inclusion criteria were as follows: (1) age > 18 years; (2) histological diagnosis of nasopharyngeal or oropharyngeal carcinoma with known HPV status (assessed by p16 immunohistochemistry and/or in situ hybridisation); (3) treatment with radical-intent IMRT between 2007 and 2017; (4) minimum follow-up of 24 months; (5) presence of dental metallic implants causing streaking artifacts in the planning kVCT severe enough to require tMVCT-based planning; (6) availability of paired kVCT and tMVCT images with acceptable registration quality. Exclusion criteria were as follows: prior RT to the head and neck region; absence of tMVCT acquisition prior to treatment; and image quality precluding reliable registration.

Of the 19 patients, 16 (10 in the test set and six in the validation set of the MAR-DTN model) were entirely independent of model training. The remaining three patients were part of the training set used to optimize the network weights for image synthesis quality. Their inclusion in the dosimetric validation does not constitute data leakage, as the network was trained to minimize pixel-level image differences, whereas the present study evaluates a downstream dosimetric task that was not part of the training objective. We verified that excluding these three patients does not alter the conclusions.

**Figure 1:**
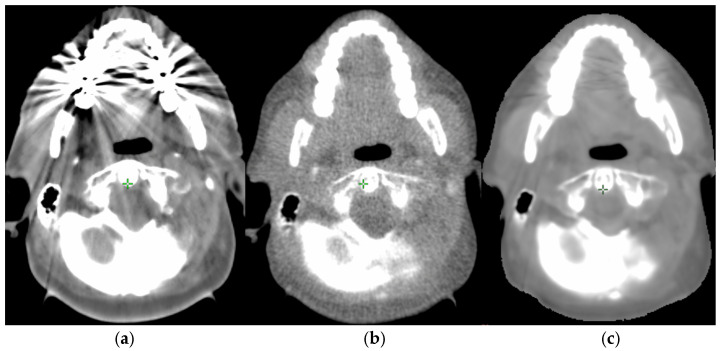
Original artifact-affected kVCT (**a**), tMVCT acquired for management of metal artifacts (**b**), and sMVCT synthesized from the planning kVCT (**c**). All three panels are displayed with the same window settings (W/L = 600/0 HU).

Patients were scanned using a CT scanner (Aquilion LB, Canon Medical Systems, Otawara, Japan) with spatial resolution of 0.98 × 0.98 or 1.17 × 1.17 mm^2^ (in-plane) and 2 mm slice thickness, at 120–135 kV tube voltage. All patients presented with dental metallic implants—including fillings and crowns composed of high atomic number (Z) materials located in the mandible and/or maxilla—whose presence caused severe streaking artifacts in the pre-treatment kVCT (Figure 1a). Of the 19 patients, 17 presented with bilateral implants, one with right-sided implants only, and one with left-sided implants only. Artifact severity was independently graded by two observers as mild in three patients (16%), moderate in nine (47%), and severe in seven (37%), with perfect inter-observer agreement for both laterality and severity (Cohen’s κ = 1.0). Tumor subsites included oropharynx (*n* = 12), base of tongue (*n* = 3), tonsil (*n* = 2), and nasopharynx (*n* = 2).

For RT treatment planning and dose calculation, a tMVCT (Figure 1b) was acquired at the treatment machine prior to each treatment course, using the image guidance system of the Hi-Art II Tomotherapy system (Accuray Inc., Sunnyvale, CA, USA), which employs a nominal energy in the imaging modality of 3.5 MV and an arc-shaped xenon detector [17]. The images were acquired with a 512 × 512 pixel matrix, and voxel size of 0.754 × 0.754 mm^2^. Pitch was 4 mm/gantry rotation and reconstruction slice thickness was 2 mm.

Treatment plans had a prescribed dose of 70.95 Gy in 33 fractions of 2.15 Gy per fraction. Plans included seven to nine, six MV IMRT fields. Treatment planning system calculated the dose on tMVCT by the Analytical Anisotropic Algorithm (AAA), version 1610, with 2.5 mm grid size [18].

### 2.2. Synthetic Images

The planning kVCT images were exported from the Eclipse Treatment Planning System 16.1 (Varian, Palo Alto, CA, USA) and were anonymized by Dicom Cleaner (PixelMed Publishing, Bangor, PA, USA) freeware software. This tool performs de-identification of patient data, including name, date of birth, treatment date, institution, accelerator type, and DICOM unique identifiers. This was necessary to prevent conflicts when later re-importing images in Eclipse after conversion into sMVCT.

The kVCT images were then converted to sMVCT (Figure 1c) using a neural network called MAR-DTN, a deep learning network capable of generating artifact-free MVCT images from kVCT images, whose architecture and training are described in detail elsewhere [16]. Briefly, an UNet-inspired architecture is employed with skip connections. The encoder progressively reduces spatial resolution to extract hierarchical features, while the decoder reconstructs the image at the original resolution using skip connections that preserve spatial detail. The network comprises approximately 1.88 million trainable parameters; to enforce artifact correction, loss function weights of up to 100 were applied in the metal artifact regions during training [16].

After importing the sMVCT into Eclipse, rigid image registration with tMVCT was performed by two medical physicists, each with at least three years of experience in RT image registration in Eclipse. A standardized protocol was followed: initial manual alignment based on bony anatomy of the cervical spine was performed, followed by automated refinement using the Eclipse rigid registration tool, which employs mutual information as a similarity measure and a Downhill simplex optimizer. Registration quality was verified visually by both operators; in cases of discrepancy, a consensus alignment was adopted.

**Figure 2:**
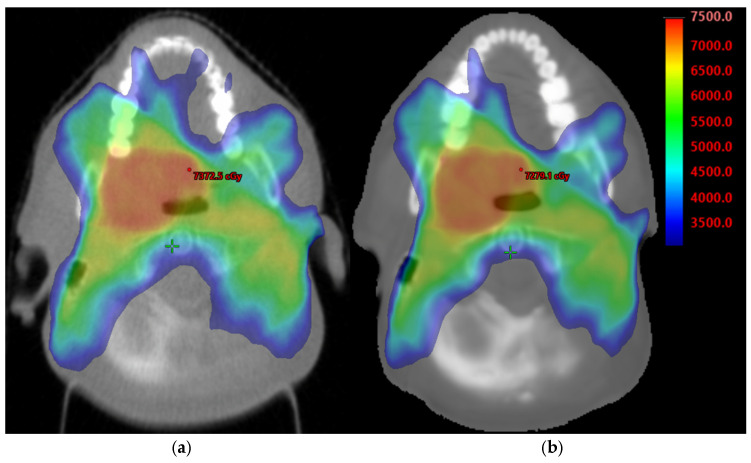
Comparison between dose distributions calculated on tMVCT (**a**) vs. sMVCT (**b**). Isodose colors are shown in the colorbar in cGy units.

The contours of target GTV and PTV70 and following OARs thyroid, parotids, brainstem, spinal cord were propagated to sMVCT to compare mean HU values between the structures in the two image types. Then, the clinical plan originally optimized on tMVCT was subsequently transferred to the sMVCT coordinate space using the rigid registration. For the electron-density map in dose calculations, the tMVCT was calibrated using the HU-to-electron-density curve acquired on the Hi-Art II Tomotherapy. The same electron-density curve was applied to the sMVCT. The dose distribution was recalculated for the plan in the sMVCT using the AAA_1610 algorithm (2.5 mm grid) without modifying the original treatment plan parameters (Figure 2).

### 2.3. HU and Dosimetric Comparison

To perform a dosimetric comparison between the two image sets, we collected dose metrics from the dose–volume histograms (DVHs) for both plans of the most relevant target structures and OAR tissues of the testing patients already listed in the previous paragraph. The metrics collected included the maximum dose received by at least 0.1 cc of brainstem [19] and the spinal cord, mean dose received by parotids [20], percentage of thyroid volume that received 45 Gy [21], the minimum dose received by the hottest 1% of volume of GTV and PTV70, and the dose covering 95% of PTV70 and GTV [22].

Pairwise differences were calculated for mean HU and specific dosimetric indices for each patient and for all structures. Pairwise Wilcoxon *p* test was done to evaluate the statistical significance of the metric differences between tMVCT and sMVCT at a 0.05 significance level. Results are reported in Table 1.

**Figure 3:**
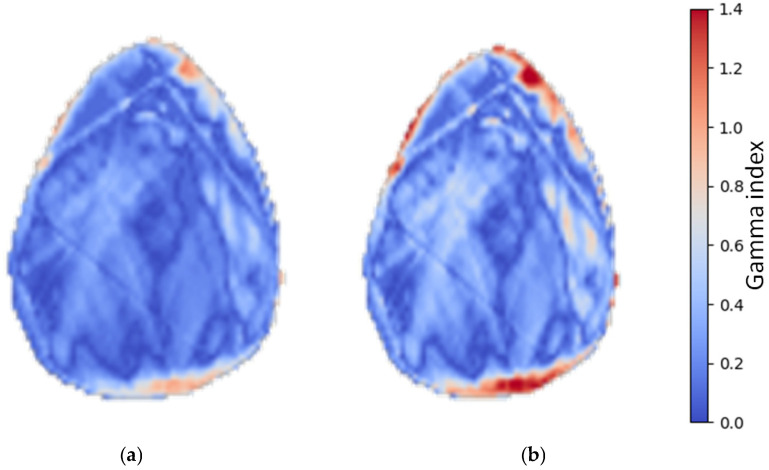
Gamma distribution for one typical case, with 3%, 3 mm (**a**) and 2%, 2 mm (**b**) criteria, respectively.

Datasets consisting of tMVCT, sMVCT and their registration were exported from Eclipse in DICOM format and imported into a Python (version 3.12) script, which is shared on Github [23]. This tool loads the dose distributions, loads and applies the rigid registration to the dose distributions, and calculates the gamma index using the library PyMedPhys, version 0.41.0 [24]. In the gamma calculation, the dose distribution recalculated on sMVCT was used as the evaluation image, while the dose distribution calculated on tMVCT served as the reference. Two sets of gamma criteria were applied: 2 mm, 2% and 3 mm, 3%, with 5% lower dose threshold for gamma calculation (Figure 3). For each criterion, the mean gamma pass rate across all patients was computed, and the 2.5th and 97.5th percentiles were reported to characterize the distribution of results.

Statistical equivalence between sMVCT and tMVCT was assessed using the Two One-Sided Tests (TOST) procedure, which evaluates equivalence by testing two complementary one-sided null hypotheses (mean difference ≤ −δ and mean difference ≥ +δ); rejection of both at the α = 0.05 level is operationally equivalent to demonstrating that the 90% confidence interval of the mean difference lies entirely within the pre-specified equivalence margin ±δ. Equivalence margins were set on clinical grounds: ±20 HU for HU comparison, corresponding to a 2% deviation in physical density (1 HU ≈ 0.1% of water density), and ±2% of the prescribed dose (±142 cGy of 70.95 Gy) for all DVH metrics, both well below the 3% accuracy threshold conventionally adopted for radiotherapy dose calculation. For the 3D gamma pass rate, a one-sided non-inferiority t-test was used against the clinically accepted thresholds of 90% (2 mm/2%) and 95% (3 mm/3%); non-inferiority was declared when the lower one-sided 95% CI of the mean pass rate exceeded the threshold.

A pre-specified sensitivity analysis was performed to assess the robustness of the conclusions to the partial overlap between the validation cohort and the MAR-DTN training set. All HU and dosimetric analyses were repeated on the strictly independent sub-cohort (*n* = 16, comprising the patients drawn from the test and validation sets of the original MAR-DTN model), and the results were compared with those of the full cohort (*n* = 19).

## 3. Results

The HU comparison between sMVCT and tMVCT showed excellent agreement across all evaluated structures (Table 1). Pairwise differences in mean HU were small for all organs, ranging from −1.1 HU (brainstem) to 9.0 HU (GTV), and none reached statistical significance (all Wilcoxon *p* > 0.05). The largest differences were observed in the GTV and PTV70, where mean HU values were slightly less negative on sMVCT than on tMVCT (−21.3 vs. −30.3 HU for GTV; −15.9 vs. −24.3 HU for PTV). In all cases, the pairwise differences remained well within clinically acceptable limits. The dosimetric comparison between dose distributions calculated on sMVCT and tMVCT is summarized in Table 1 and results of gamma calculations are in Table 2.

TOST equivalence analysis (margin ±20 HU) confirmed statistical equivalence between sMVCT and tMVCT for all seven structures (TOST *p* < 0.05; 90% CI within ±20 HU; maximum *p* = 0.0136 for PTV70). Sensitivity analysis on the strictly independent sub-cohort (*n* = 16) yielded smaller mean differences than the full cohort across all structures (e.g., thyroid +5.20 → +1.51 HU; spinal cord +5.75 → +1.30 HU; GTV +3.22 → +1.62 HU) and confirmed equivalence for all structures (TOST *p* < 0.05). Wilcoxon signed-rank tests yielded *p* > 0.05 for all comparisons in both cohorts.

All nine DVH metrics achieved formal dosimetric equivalence between sMVCT and tMVCT (TOST *p* < 0.05 with margin ±2% of prescription) in both the full cohort (*n* = 19) and the sensitivity sub-cohort (*n* = 16) (full results in Table S1, Supplementary Materials). All mean differences were below 2% of the prescribed dose. The largest systematic difference was observed for PTV70 D1% (+0.7% of prescription on average), discussed below. It should be noted that the Wilcoxon and TOST tests address distinct hypotheses: the Wilcoxon test detects any directional difference, while TOST evaluates whether the magnitude of the difference is contained within a pre-specified clinically relevant margin. The two outcomes for PTV70 D1%—Wilcoxon *p* < 0.001 alongside TOST equivalence within ±2%—are therefore not contradictory: a small but systematic shift exists, but it is well below the threshold of clinical relevance. The 3D gamma pass rates were 94.31 ± 2.54% (mean ± SD) for the 2 mm/2% criterion and 97.57 ± 1.40% for the 3 mm/3% criterion (*n* = 19); both passed formal non-inferiority tests against the clinically accepted thresholds of 90% and 95%, respectively (one-sided *p* < 0.0001). In one patient, the 2 mm/2% pass rate fell slightly below the 90% threshold (88.7%); in clinical practice, such individual cases would be flagged for review by the medical physicist before plan delivery, in line with standard QA workflow for AI-based imaging tools. Sensitivity analysis on *n* = 16 yielded slightly higher pass rates (94.92% and 97.89%, respectively), confirming robustness of the gamma analysis.

## 4. Discussion

Artifacts from metal objects in CT, besides complicating target volume delineation, can adversely impact radiation therapy treatment planning by compromising the accuracy of dose calculation. In our results, sMVCT could successfully remove metal artifacts, leading to clinically useful datasets for dosimetric purposes [25].

Should prospective validation confirm these findings, the proposed clinical workflow for integration of sMVCT into RT planning would be as follows. For patients with dental metallic implants requiring head and neck IMRT, the planning kVCT is acquired as per standard practice and converted to sMVCT using MAR-DTN. The sMVCT image is then imported into the treatment planning system and used as the primary planning image for target and OAR delineation, dose calculation, and plan optimization. This approach provides the dosimetric accuracy of MVCT-based planning without requiring a dedicated tMVCT acquisition session on the Tomotherapy machine, which is only available at centers equipped with this specific technology, thereby making artifact-robust planning accessible also to centers using conventional linacs. The dosimetric accuracy demonstrated in this study supports the clinical feasibility of this workflow. These results support the dosimetric feasibility of sMVCT-based planning under the conditions of this study, while clinical adoption requires confirmation in independent multicentre prospective cohorts.

As described in the Introduction, dental artifacts arise from beam hardening and photon starvation at high-Z implants, and conventional MAR algorithms, although effective in reducing streak artifacts, are known to introduce secondary artifacts and to distort tissue density near metallic objects [11,26]. These limitations directly affect HU accuracy and, consequently, dose calculation reliability. The sMVCT approach evaluated in this study avoids both the original artifacts and the limitations of interpolation-based MAR by using the MV energy domain, where metal attenuation is drastically reduced, while preserving the spatial resolution and soft tissue information present in the planning kVCT source image.

The clinical utility of synthetic images is inherently dependent on the accuracy and reliability of the generated images, underscoring the importance of rigorous image quality evaluation [27]. The evaluation of synthetic images is commonly performed using metrics such as Peak Signal-to-Noise Ratio (PSNR), which describes pixel-level differences between synthetic and real image and Structural Similarity Index Measure (SSIM) which combines evaluation of the luminance, contrast, and structure in two moving windows in the synthetic and reference image, applied in a way similar to the convolution operation [28]. Besides the drawbacks inherent to each metric, these only reflect the perceived visual similarity and pixel difference. They do not account for physical adherence of images and do not account for possible hallucinations that, though small in the accepted metrics, could result in significant errors in the propagation in postprocessing techniques such as automatic segmentation or dose calculation.

For these reasons, we analyzed HU values, which represent tissue density, where water is defined as 0 HU and air as −1000 HU; a 1 HU difference corresponds to a 0.1% change relative to the density of water. For this reason, validation of dose calculation accuracy on synthetic images intended for RT planning is essential. In our results, the pairwise differences in mean HU were small (all within 10 HU) for all structures and patients; none reached statistical significance. For comparison, Singhrao et al. obtained similar differences in soft tissue [29]. In assessing dose calculation on HyperSight kV-CBCT imaging system, Sijtsema found that with HU errors within 35 HU, prostate maximum deviation in PTV mean dose was 1.1% [30], indicating that HU uncertainty should contribute to our dose calculation error for less than 1%.

A small but systematic positive bias in D1% of target volumes (median +0.7% of prescription for PTV70 D1%, well within the TOST equivalence margin of ±2%) was observed in the dosimetric comparison. This is at least partly attributable to the higher stochastic noise of tMVCT (Figure 1b) acquisitions compared with sMVCT, which is intrinsically smoothed by the deep learning network (see Figure 1c). This interpretation is supported by significantly higher HUmax values in the GTV on tMVCT compared with sMVCT (median difference +68 HU, Wilcoxon *p* = 0.020) and a similar trend in PTV70 (+41 HU, *p* = 0.083). The neural network’s regularization removes outlier high-density voxels in target volumes that locally over-attenuate the dose in tMVCT, slightly reducing tMVCT-calculated D1%. Importantly, this small bias does not impact target coverage (D95% differences below 0.5% in all cohorts) nor OAR sparing (Dmean and D0.1cc differences within 1.5% of prescription). The gamma pass rate of 97.6 (95%CI: 94.8–99%), and 94.3 (95%CI: 89.3–97.1) for (3%, 3 mm) and (2%, 2 mm) criteria, respectively, that we obtained are consistent with results by Singhrao for synthetic CTs (sCT) of head and neck versus true CT, where gamma rates of 95.5 ± 2% (3 mm, 3%) and 92.7 ± 2.1% (2 mm, 2%) were obtained [29]. Head and neck results appear lower than other sites such as, for instance, the 99.76% (3%, 3 mm) and 97.25% (2%, 2 mm) reported for MRI-based sCT of the brain only [31]. It should be noted that gamma pass rate for dose calculation on synthetic images may be influenced by the treatment site. For MRI-derived sCTs of pelvis, gamma pass rates of 95 ± 2% and 92 ± 3% for prostate and rectum treatment plans, respectively, were reported [32]. In conclusion, our results of gamma pass rate fall within the range of results in the previous works on synthetic imaging for dose calculation and agree well with results on the head and neck.

The spatial distribution of gamma failures in Figure 3 reveals that elevated gamma values are concentrated at the patient surface rather than in the central dose region, suggesting that residual dose discrepancies are not attributable to errors in HU conversion or dose calculation per se, but rather to slight geometric misalignments between the kVCT used to generate the sMVCT and the tMVCT acquisition. Such misalignments are an inherent consequence of the separate imaging sessions: the planning kVCT and the tMVCT are acquired at different times and on different machines, with the patient repositioned between sessions. Head and neck patients are particularly susceptible to inter-session geometric variability due to differences in neck curvature and head tilt. This positional uncertainty likely accounts for the slightly lower gamma pass rates observed in our cohort compared with studies of other anatomical sites, where such inter-modality registration errors are less pronounced.

The main novelty of this study lies in the clinical dosimetric validation of a deep learning domain transformation approach for generating sMVCT from artifact-affected kVCT, evaluated against physically acquired tMVCT as the clinical ground truth. Unlike prior sinogram-based MAR methods that use MVCT as a prior image for kVCT correction [14,15], our approach produces a standalone sMVCT image that can be used directly for treatment planning, without requiring a separate MVCT acquisition at the time of plan generation. This study has several limitations. First, the retrospective single-institution design and the small cohort size (*n* = 19) limit generalisability; future multicentre studies with larger and more heterogeneous populations are warranted. Second, rigid image registration was used rather than deformable image registration (DIR). While DIR is in principle better suited to account for soft tissue deformations between the kVCT and tMVCT acquisitions in the head and neck region, in the specific context of the present study, rigid registration may be more robust than DIR: intensity-based deformable algorithms are known to be locally destabilized by image artifacts such as dental metal artifacts in head and neck CT, which generate anomalous HU regions that propagate unreliable similarity-metric gradients and can produce spurious deformation vectors [33]. A rigid alignment, which relies on global bony anatomy, is therefore less sensitive to artifact-induced local registration failures in this specific clinical scenario. Third, a direct dosimetric comparison between sMVCT- and MAR-corrected kVCT was not performed, as this was beyond the scope of the present validation study. Fourth, results are specific to the AAA algorithm and the Tomotherapy platform; validation with other dose calculation algorithms and linac-based MVCT systems would strengthen generalisability. Fifth, although a complete sensitivity analysis confirmed that the conclusions of this study are robust to the exclusion of three patients that were part of the MAR-DTN training set (HU and DVH differences smaller in the strictly independent sub-cohort for the structures with the largest mean differences and comparable for the others; all TOST equivalence and gamma non-inferiority tests confirmed in both cohorts), we acknowledge that, in a small cohort such as this, the partial overlap between training and validation populations represents a residual methodological limitation.

QA of AI is a crucial step for the safe application of new technologies to medicine [34]. Recent ESTRO/AAPM guidelines recommend procedures for periodic QA of the AI tool before clinical introduction (highly recommended) as well as for upgrades and updates before clinical re-introduction (highly recommended) [27]. In practice, however, QA methodologies for AI-based systems in RT are still an emerging area, and standardized protocols are not yet well established [35]. Phantoms could assist routine quality control of sCTs by providing quality controls in reproducible settings, like how they are used for the QA of CT scans. However, this approach may be challenging for AI systems that have been primarily trained on patient data and may fail on non-anthropomorphic phantoms. The combined HU comparison and gamma index analysis presented here provides a basis for a practical dosimetric validation framework for AI-based synthetic imaging tools in RT, suitable as the foundation of a QA protocol prior to clinical introduction and application in line with current ESTRO/AAPM recommendations.

## 5. Conclusions

sMVCT-based planning shows dosimetric consistency with tMVCT under the conditions of this study, providing a promising alternative for centers without access to tMVCT and a foundation for future prospective evaluation in larger and more heterogeneous cohorts. Pairwise differences in mean HU values across all evaluated structures were small (≤10 HU) and statistically non-significant, indicating that the synthetic images provide an accurate representation of tissue densities despite the presence of metal artifacts in the source kVCT. Dose distributions recalculated on sMVCT showed excellent agreement with those on tMVCT. The small residual disagreements, predominantly located at the patient surface, are attributable to repositioning differences between the two acquisitions rather than to systematic errors of the synthetic image generation method. These results support the dosimetric feasibility of sMVCT-based planning as an alternative to tMVCT for patients with dental implants, eliminating the need for a dedicated MV imaging session, while clinical adoption requires confirmation in larger prospective cohorts. The evaluation framework presented here provides a basis for a practical quality assurance framework for AI-based synthetic image tools in RT treatment planning, should prospective validation confirm these findings.

## Figures and Tables

**Table 1:** Comparison of HUs and dosimetric results of tMVCT and sMVCT.

Organ	Metric, Units	Average tMVCT	Average sMVCT	Pairwise Difference	Pairwise Wilcoxon *p*
Thyroid	Mean HU	40.2	45.4	+5.2	0.26
	V45Gy, %	38.5	39.2	+0.7	0.40
Left Parotid	Mean HU	9.9	11.0	+1.0	0.20
	Mean Dose, cGy	3058.2	3044.0	−14.1	0.40
Right Parotid	Mean HU	5.4	8.3	+3.0	0.23
	Mean dose, cGy	3113.8	3087.4	−26.4	0.29
Brainstem	Mean HU	40.8	39.7	−1.1	0.37
	D0.1cc, cGy	3804.8	3826.6	+21.8	0.68
Spinal Cord	Mean HU	50.3	56.0	+5.8	0.28
	D0.1cc, cGy	3733.0	3718.2	−14.9	0.65
GTV	Mean HU	−5.9	−2.7	+3.2	0.10
	Minimum D95%, cGy	7097.1	7069.1	−28.0	0.83
	Maximum D1%, cGy	7347.3	7367.5	+20.1	0.01
PTV	Mean HU	−24.3	−15.9	+8.4	0.09
	Minimum D95%, cGy	7024.0	7011.8	−12.2	0.20
	Maximum D1%, cGy	7344.9	7397.7	+52.8	<0.001

**Table 2:** Comparison of γ index for two metrics: (3 mm/3%), (2 mm/2%) and 95% percentiles.

Statistic	γ Index (3 mm/3%)	γ Index (2 mm/2%)
Average	97.6	94.3
Percentile (2.5%)	94.8	89.3
Percentile (97.5%)	99.0	97.1

## Data Availability

The code for gamma calculations is shared in Ref. [23].

## Supplementary Materials

The following supporting information can be downloaded at https://www.mdpi.com/article/10.3390/cancers18101603/s1, Table S1: Formal equivalence testing (Two One-Sided Tests, TOST) for HU and DVH metrics, and one-sided non-inferiority test for the 3D gamma pass rate against clinically accepted thresholds, comparing sMVCT and tMVCT. Equivalence margins: ±20 HU for HU comparisons (≈2% of physical density); ±5% for V45 thyroid; ±2% of prescribed dose (±142 cGy of 70.95 Gy) for all dose metrics. Non-inferiority thresholds for the gamma pass rate were 90% (2 mm/2%) and 95% (3 mm/3%). Each metric is reported twice: once for the full cohort (*n* = 19, “full”) and once for the strictly independent sub-cohort, excluding three patients drawn from the MAR-DTN training set (*n* = 16, “sensitivity”). Equivalence is declared when the 90% CI of the mean difference lies entirely within the margin (TOST *p* < 0.05); non-inferiority is declared when the lower one-sided 95% CI of the gamma pass rate exceeds the threshold.

## Abbreviations

The following abbreviations are used in this manuscript:

AAA	Analytical Anisotropic Algorithm
CBCT	Cone-Beam Computed Tomography
CI	Confidence Interval
DICOM	Digital Imaging and Communications in Medicine
DIR	Deformable Image Registration
DVH	Dose–Volume Histogram
GAN	Generative Adversarial Network
GTV	Gross Tumor Volume
HPV	Human Papillomavirus
HU	Hounsfield Unit
IGRT	Image-Guided Radiotherapy
IMRT	Intensity-Modulated Radiation Therapy
kVCT	Kilovoltage Computed Tomography
kV-CBCT	Kilovoltage Cone-Beam Computed Tomography
MAR	Metal Artifact Reduction
MAR-DTN	Metal Artifact Reduction through Domain Transformation Network
MRI	Magnetic Resonance Imaging
MVCT	Megavoltage Computed Tomography
OAR	Organ at Risk
PSNR	Peak Signal-to-Noise Ratio
PTV	Planning Target Volume
QA	Quality Assurance
RT	Radiation Therapy
sMVCT	Synthetic Megavoltage Computed Tomography
SSIM	Structural Similarity Index Measure
tMVCT	True Megavoltage Computed Tomography
TOST	Two one-sided tests equivalence testing
TPS	Treatment Planning Software
