# Supplementary material for: Validation of Synthetic Megavoltage Computed Tomography (MVCT) for Dose Calculation in Radiotherapy Treatment Planning
Source: Cancers (Basel). 2026 May 14;18(10):1603. doi: 10.3390/cancers18101603 (PMC13204460; doi:10.3390/cancers18101603)
Supplement: Supplementary file 1 [file cancers-18-01603-s001.zip › cancers-4240892-supplementary.pdf]

Table S1 — Formal equivalence (TOST) and sensitivity analysis

Table S1. Formal equivalence testing (Two One-Sided Tests, TOST) for HU and DVH metrics, and one-sided non-inferiority test for the 3D gamma pass rate against clinically accepted thresholds, comparing sMVCT and tMVCT. Equivalence margins: ±20 HU for HU comparisons (≈ 2% of physical density); ±5% for V45 thyroid; ±2% of prescribed dose (±142 cGy of 70.95 Gy) for all dose metrics. Non-inferiority thresholds for the gamma pass rate were 90% (2 mm/2%) and 95% (3 mm/3%). Each metric is reported twice: once for the full cohort (n = 19, "full") and once for the strictly independent sub-cohort, excluding three patients drawn from the MAR-DTN training set (n = 16, "sensitivity"). Equivalence is declared when the 90% CI of the mean difference lies entirely within the margin (TOST $p < 0.05$); non-inferiority is declared when the lower one-sided 95% CI of the gamma pass rate exceeds the threshold.

Metric	Margin	n	Mean diff. (sMV – tMV)	90% CI	TOST p	Equivalent?
**HU comparison (margin ±20 HU)**						
Thyroid HU	±20 HU	19	+5.20	[-1.77, +12.18]	0.0009	✓
Thyroid HU (sensitivity, n=16)	±20 HU	16	+1.51	[-3.67, +6.69]	<0.0001	✓
Right parotid HU	±20 HU	19	+2.96	[-0.79, +6.70]	<0.0001	✓
Right parotid HU (sensitivity, n=16)	±20 HU	16	+0.56	[-2.80, +3.91]	<0.0001	✓
Left parotid HU	±20 HU	19	+1.05	[-7.49, +9.59]	0.0006	✓
Left parotid HU (sensitivity, n=16)	±20 HU	16	-2.74	[-11.87, +6.39]	0.0024	✓
Brainstem HU	±20 HU	19	-1.13	[-2.96, +0.70]	<0.0001	✓
Brainstem HU (sensitivity, n=16)	±20 HU	16	-1.51	[-3.55, +0.52]	<0.0001	✓
Spinal cord HU	±20 HU	19	+5.75	[-1.03, +12.54]	0.0009	✓
Spinal cord HU (sensitivity, n=16)	±20 HU	16	+1.30	[-4.46, +7.06]	<0.0001	✓
GTV HU	±20 HU	19	+3.22	[-2.40, +8.84]	<0.0001	✓
GTV HU (sensitivity, n=16)	±20 HU	16	+1.62	[-4.53, +7.78]	<0.0001	✓
PTV70 HU	±20 HU	19	+8.41	[+0.05, +16.78]	0.0136	✓
PTV70 HU (sensitivity, n=16)	±20 HU	16	+7.21	[-2.56, +16.97]	0.0182	✓
**Dosimetric comparison (margin ±2% of prescription = ±142 cGy; ±5% for V45 thyroid)**						
V45 thyroid (%)						
V45 thyroid (%) (sensitivity, n=16)	±5%	16	-0.44	[-1.17, +0.28]	<0.0001	✓
Dmean R parotid (cGy)	±142 cGy	19	-26.38	[-73.98, +21.21]	0.0003	✓
Dmean R parotid (cGy) (sensitivity, n=16)	±142 cGy	16	-46.50	[-93.07, +0.07]	0.0013	✓
Dmean L parotid (cGy)	±142 cGy	19	-14.14	[-80.69, +52.41]	0.0019	✓
Dmean L parotid (cGy) (sensitivity, n=16)	±142 cGy	16	-50.16	[-100.67, +0.36]	0.0031	✓
D0.1cc brainstem (cGy)	±142 cGy	19	+21.79	[-33.86, +77.45]	0.0007	✓
D0.1cc brainstem (cGy) (sensitivity, n=16)	±142 cGy	16	-8.20	[-58.02, +41.62]	0.0001	✓
D0.1cc spinal cord (cGy)	±142 cGy	19	-14.86	[-85.36, +55.64]	0.0029	✓

D0.1cc spinal cord (cGy) (sensitivity, n=16)	±142 cGy	16	-29.62	[-112.14, +52.90]	0.0153	✓
GTV D95% (cGy)	±142 cGy	19	-28.05	[-82.34, +26.25]	0.0009	✓
GTV D95% (cGy) (sensitivity, n=16)	±142 cGy	16	-31.44	[-96.69, +33.82]	0.0048	✓
GTV D1% (cGy)	±142 cGy	19	+20.15	[-5.59, +45.88]	<0.0001	✓
GTV D1% (cGy) (sensitivity, n=16)	±142 cGy	16	+12.29	[-16.02, +40.59]	<0.0001	✓
PTV70 D95% (cGy)	±142 cGy	19	-12.22	[-30.45, +6.02]	<0.0001	✓
PTV70 D95% (cGy) (sensitivity, n=16)	±142 cGy	16	-18.58	[-37.03, -0.13]	<0.0001	✓
PTV70 D1% (cGy)	±142 cGy	19	+52.84	[+40.48, +65.20]	<0.0001	✓
PTV70 D1% (cGy) (sensitivity, n=16)	±142 cGy	16	+48.98	[+37.06, +60.90]	<0.0001	✓

Gamma index—one-sided non-inferiority test

**Metric**	**Threshold**	**n**	**Mean (%)**	**One-sided 95% CI lower bound**	**p**	**Non-inferior?**
γ 2 mm/2%	90%	19	94.31	93.30%	<0.0001	✓
γ 2 mm/2% (sensitivity, n=16)	90%	16	94.92	94.04%	<0.0001	✓
γ 3 mm/3%	95%	19	97.57	97.02%	<0.0001	✓
γ 3 mm/3% (sensitivity, n=16)	95%	16	97.89	97.37%	<0.0001	✓
